# GTP-binding facilitates EB1 recruitment onto microtubules by relieving its auto-inhibition

**DOI:** 10.1038/s41598-018-28056-y

**Published:** 2018-06-28

**Authors:** K. K. Gireesh, A. Shine, R. Bhagya Lakshmi, Vinesh Vijayan, Tapas K. Manna

**Affiliations:** 1 School of Biology, Indian Institute of Science Education and Research Thiruvananthapuram, CET Campus, Thiruvananthapuram, 695016 Kerala India; 2 School of Chemistry, Indian Institute of Science Education and Research Thiruvananthapuram, CET Campus, Thiruvananthapuram, 695016 Kerala India

## Abstract

Microtubule plus end-binding protein, EB1 is a key regulator of microtubule dynamics. Auto-inhibitory interaction in EB1 has previously been shown to inhibit its ability to bind to microtubules and regulate microtubule dynamics. However, the factors that promote its microtubule regulatory activity by over-coming the auto-inhibition are less known. Here, we show that GTP plays a critical role in promoting the microtubule-targeting activity of EB1 by suppressing its auto-inhibition. Our biophysical data demonstrate that GTP binds to EB1 at a distinct site in its conserved N-terminal domain. Detailed analyses reveal that GTP-binding suppresses the intra-molecular inhibitory interaction between the globular N-terminus and the C-terminal coiled-coil domain. We further show that mutation of the GTP-binding site residues in N-terminus weakens the affinity for GTP, but also for the C-terminus, indicating overlapping binding sites. Confocal imaging and biochemical analysis reveal that EB1 localization on the microtubules is significantly increased upon mutations of the GTP-binding site residues. The results demonstrate a unique role of GTP in facilitating EB1 interaction with the microtubules by relieving its intra-molecular inhibition. They also implicate that GTP-binding may regulate the functions of EB1 on the cellular microtubules.

## Introduction

Polymerization and depolymerization dynamics of microtubules play crucial roles in cell division, cell motility and cell morphogenesis. The plus ends of microtubules are the key binding sites of a wide array of regulatory proteins that modulate dynamics and mediate microtubule interactions with various sub-cellular structures. Interestingly, it has been found that functions of some of the important microtubule regulators are inhibited by intra-molecular interactions within their structures. For example, the microtubule-tracking activities of motor proteins of Kinesin 1–3 families are auto-inhibited by their C-termini^1–5^ and such auto-inhibition seems to be crucial for certain developmental processes. In particular, relieving the auto-inhibition in Kinesin-1 in *Drosophila* has been shown to be very critical for the growth of the organism to adulthood^6^. A few recent studies have reported that microtubule plus end tracking proteins (+TIPs) exhibit auto-inhibition against their microtubule regulatory activities. For example, the microtubule regulatory activity of a central component of the +TIPs family proteins, EB1, has been shown to be inhibited through an intra-molecular interaction between its key structural domains^7,8^. Similarly, intra-molecular inhibition in another +TIP, CLIP-170 has been shown to activate specific molecular signal, such as phosphorylation to modulate its microtubule regulatory activity^9,10^. Factors that control auto-inhibition in +TIPs are less known.

EB1 is a conserved member of the +TIPs family that can autonomously track the microtubule plus end and regulate plus end dynamics^11–16^. It also orchestrates recruitment other +TIPs to the plus end and stabilizes assembly of the +TIPs complexes at the end^17–21^. EB1 is a dimeric protein consisting of a globular N-terminus, an unstructured linker region, and an extended C-terminal region. The C-terminus consists of a conserved coiled-coil domain (residues 191–246), called as EB-homology (EBH) domain that is involved in the dimerization of EB1. The EBH domain and the very terminal disordered tail region (247–268) are the binding sites for other +TIP proteins and are also involved in localizing those proteins to the plus end^22–24^. The N-terminus largely consists of a conserved calponin homology (CH) domain from residues 1–130. This region mainly exists as monomer^14,25^ and is crucial for microtubule binding^8,26^. Specifically, the positively charged amino acids of this domain are critical for microtubule binding^8,27,28^. The CH domain is also responsible for the autonomous plus end tracking activity of EB1^8,18,25,29^.

Previous studies have shown that EB1 C-terminus negatively regulates the microtubule binding- and assembly-promoting activity of EB1 by binding to the CH domain and countering the CH domain–microtubule interaction^7,8^. Consistent with these findings, another study has shown that the residues of the CH domain involved in the interaction between CH domain and EB1 C-terminus, largely overlap with the microtubule-binding sites of the CH domain^26^. However, the mechanism how EB1 is recruited to the microtubules by overcoming this intra-molecular inhibition remains poorly understood. Structural analyses of human recombinant EB1 and its fission yeast homolog, Mal3 have demonstrated that EB1 can recognize GTP-bound tubulin structures at the plus end^25,30^. Electron microscopy analyses have shown that Mal3 is localized at a site close to the exchangeable GTP-binding site of tubulin in the microtubule lattice^31^. Using human recombinant EB1, it has also been shown that EB1 can sense the GTP-hydrolysis-mediated conformational change in the microtubule lattice^31^. In a recent study, we have demonstrated that EB1 can also directly bind to GTP^32^. All these findings are suggestive of a GTP-dependent mechanism of EB1 regulation on the microtubules. In this work, we show that GTP-binding inhibits the intra-molecular interaction in EB1 and stimulates its localization onto microtubules. Isothermal titration calorimetry and NMR analyses reveal that GTP binds to the CH-domain of EB1 and further show that GTP-binding inhibits the auto-inhibitory interaction between the CH domain and EB1 C-terminus. Biochemical analysis and fluorescence imaging reveal that GTP-binding facilitates EB1 recruitment onto the microtubules *in vitro*. The results first time demonstrate that GTP suppresses the intra-molecular inhibition in EB1 and facilitates its association with microtubules.

## Results and Discussion

### GTP binds to the calponin homology (CH) domain of EB1 in its N-terminus

**Figure 1:**
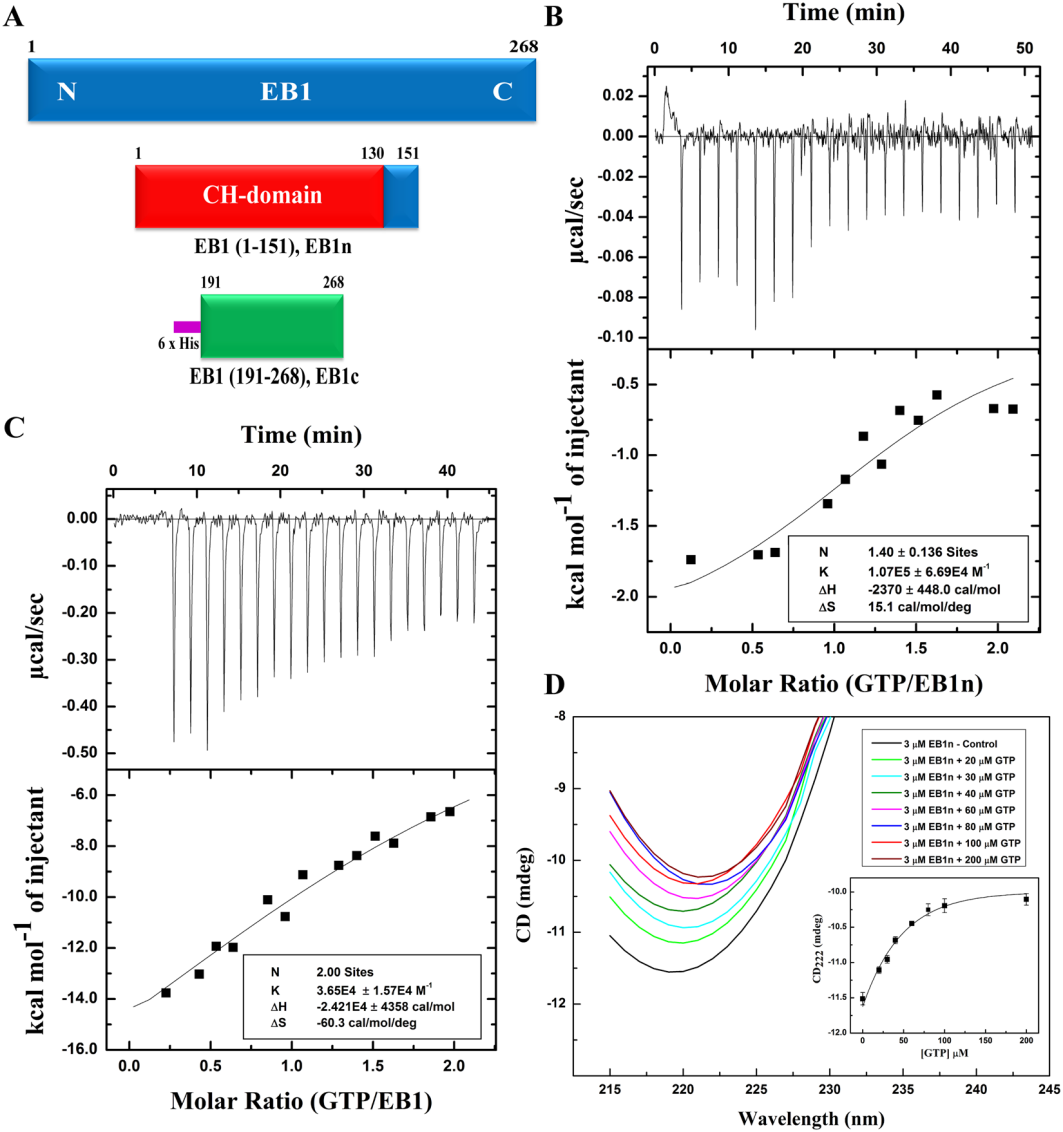
GTP binds to the N-terminus of EB1. (**A**) Schematic representations of full length EB1, EB1 N- terminus (1–151), EB1n, showing its microtubule binding calponin homology (CH) domain (1–130); 6x His tagged EB1 C-terminus (191–268), EB1c. (**B** and **C**) The upper panels show representative raw isothermal titration calorimetry (ITC) data obtained from 20 injections of 300 µM GTP to 30 µM EB1n and 200 µM GTP to 20 µM full length EB1, respectively in PEM buffer at 25 °C. Lower panel in each shows nonlinear least-square fit plot of the heat changes per mole of the added GTP in the titration as a function of the molar ratio of GTP and EB1n or full length EB1 by using Origin 7. The insets in (**B** and **C**) show the binding parameters N (stoichiometry), K (association constant), ΔH (enthalpy change), ΔS (change in entropy). The data for EB1n was fitted naturally and that of the full length EB1 were fitted at fixed N = 2.0. The data for both (**B** and **C**) represent one out of three independent experiments for each. (**D**) Far UV CD spectra of EB1n (3 μM) in the absence (black) and presence of 20 μM (green), 30 μM (cyan), 40 μM (olive), 60 μM (magenta), 80 μM (blue), 100 μM (red), and 200 μM (brown) GTP. Inset shows the plot of the ellipticity value at 222 nm against GTP concentration. Data show mean +/− SEM (three experiments).

We first determined the molecular details of EB1-GTP binding. As the N- and C-terminus of EB1 are well separated by the unstructured linker region and they individually represent independent structural domains^24,33^, we first assessed GTP-binding activity of the individual N- (residues 1–151) and C-terminus (residues 191–268) of EB1 (Fig. 1A), hereafter referred as EB1n and EB1c, respectively. Both the domains were obtained with ~100% purity (Supplementary Fig. S1A,B). ITC titration showed that GTP specifically binds to the EB1n domain (Fig. 1B). The heat change profile of the titration of EB1n with GTP showed a pattern characteristic of a binding isotherm (Fig. 1B). The reaction was predominantly enthalpy-driven with ~−2.4 +/− 0.4 Kcal/mol heat change. Data analysis also revealed ~1.4 +/− 0.2 GTP-binding sites per EB1n molecule based on natural fitting of the data. ITC titration for GTP-binding of the full length EB1 was also performed under similar condition (Fig. 1C). Both EB1n and the full length EB1 exhibited a moderate to low affinity K_d_ ranging from ~10 to ~30 µM (based on three experiments for each) (Methods). It was also observed that the EB1c region did not exhibit any characteristic binding (Supplementary Fig. S2A). We next determined how GTP-binding affects the structure of EB1n. Far-UV CD data showed a dose-dependent change of secondary structure in EB1n on addition of GTP (Fig. 1D). A significant extent (~45%) of structural change occurred at 1: 10 molar ratio of EB1n and GTP (Inset plot, Fig. 1D). The effect reached saturation at 1: ~25 molar ratio of EB1n and GTP. As expected, no structural change was observed on addition of GTP to EB1c (tary Fig. S2B).

### GTP interacts with the amino acids at a distinct site in EB1 CH domain

We next characterized the binding sites of GTP in EB1n by NMR. First, the NMR backbone resonance re-assignment^26^ was carried out using standard triple resonance NMR experiments using double labeled ^13^C-^15^N EB1n protein. We could identify around 90% of the backbone resonances of EB1n. The resonances that were missing were mostly of the unstructured region of the C-terminus of EB1n (Supplementary Fig. S3).

**Figure 2:**
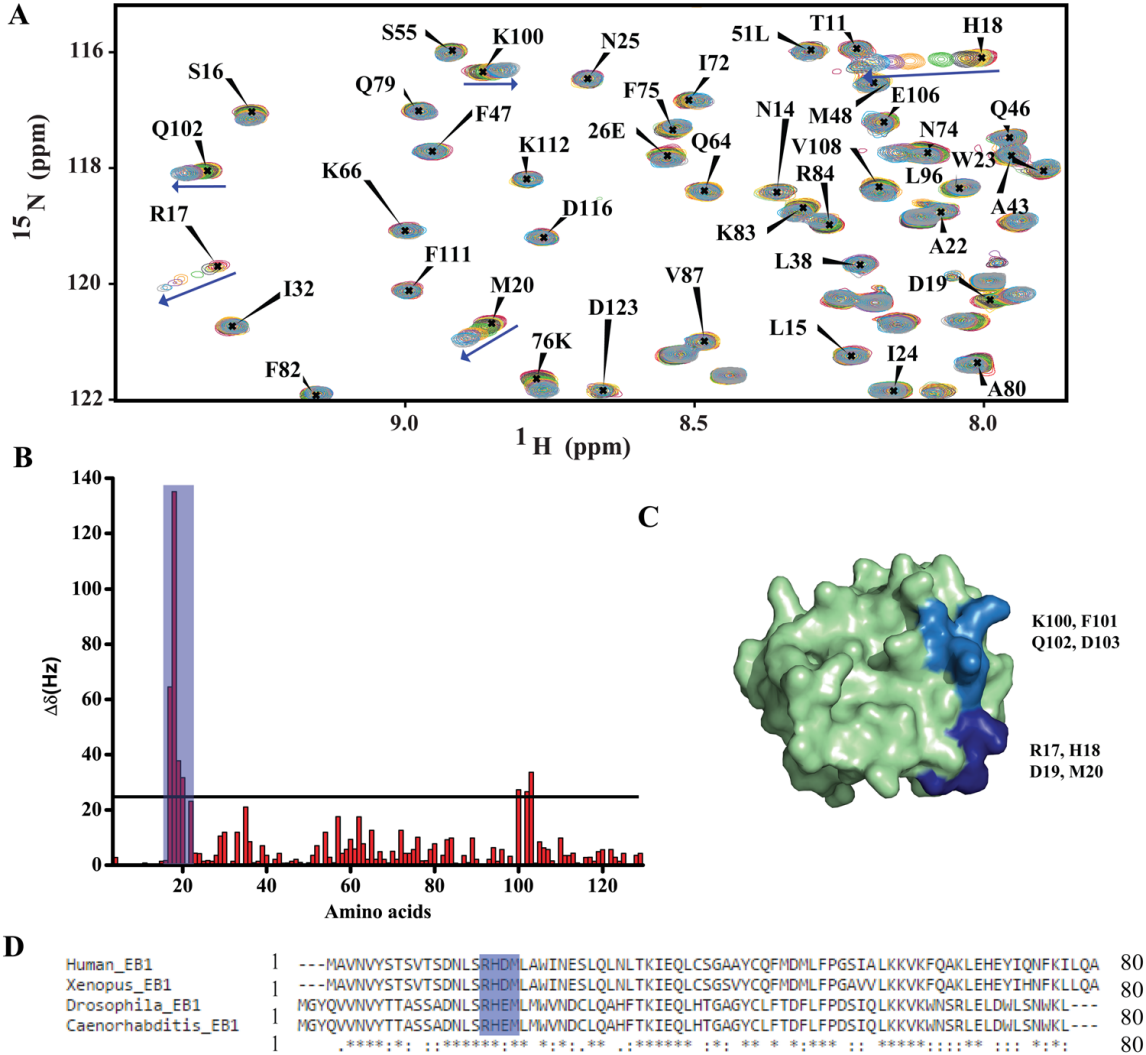
Identification of the GTP binding sites in EB1n by NMR. (**A**) Overlay of a part of 2D ^1^H-^15^N HSQC NMR spectra of EB1n with increasing concentrations of GTP (0.05 to 25 mM). The spectra were measured in 700 MHz spectrometer. The peaks of EB1n (0.1 mM) in the absence (red) and presence of increasing concentrations of GTP at 0.05 mM (maroon), 0.5 mM (pale green), 1 mM (yellow), 2 mM (grey), 5 mM (green), 10 mM (orange), 15 mM (purple), 20 mM (blue) and 25 mM GTP (light grey) are shown. The chemical shift changes of H18, R17, M20, K100 and Q102 with the addition of 25 mM GTP are shown by blue arrows. (**B**) The plot shows the combined chemical shift differences observed in ^1^H-^15^N HSQC spectra of EB1n (0.1 mM) and EB1n (0.1 mM) with 25 mM GTP of all residues. R17, H18, D19 and M20 show significantly large chemical shift perturbations compared to other amino acid residues. The stretch of amino acids K100-D103 showed a small change in the chemical shift position. A cut-off of chemical shift change 25 Hz is shown by a straight line. Change of 25 Hz is considered to be reasonable. (**C**) Three dimensional surface representation of EB1n CH domain (residues 1–130; PDB: 1PA7)^8^. The highly perturbed set of residues (R17, H18, D19 and M20) are highlighted with dark blue color, residues around K100 that showed small perturbation are shown by light blue colour. (**D**) Alignment of amino acid sequences of EB1 (1–80) in different species by using Clustal Omega. The blue colored regions show conservation of R17, H18, D19, and M20 in species, human, *Xenopus*, *Drosophila* and *C. elegans*.

To identify the amino acids of EB1n responsible for GTP binding, we performed ^1^H-^15^N HSQC (hetero-nuclear single quantum coherence) titration of EB1n with GTP at pH 6.9. The pH of the stock GTP solution that was added to the proteins was pre-adjusted to pH 6.9. Increasing concentrations of GTP (0.05 to 25 mM) were added to ^15^N- labeled EB1n and the relative changes in the peak position of HSQC spectra were monitored. The HSQC spectra of EB1n measured at different GTP concentrations showed that the chemical shifts corresponding to only a small subset of peaks in EB1n were changed with the addition of GTP (Figs 2A and S4). The majority of the peaks in the HSQC spectra remained stationary while the peaks corresponding to the residues, Arg 17 (R17), His 18 (H18), Asp 19 (D19) and Met 20 (M20) were significantly shifted with the addition of GTP. The combined chemical shift differences of all the residues of EB1n at 25 mM GTP were plotted (Fig. 2B) and the plot clearly showed that the most significantly affected residues were in the stretch from R17 to M20 (highlighted in Fig. 2B). The chemical shift of H18 had a maximum perturbation of 135 Hz followed by R17 of 64 Hz. It was apparent from the published X-ray structure (PDB-1PA7) of EB1 CH domain that the region corresponding to the most affected residues in response to GTP addition, the R17-M20 stretch, is located near the end of the first helix (Figs 2C and S5)^8^. Small chemical shift changes were also observed for peaks corresponding to residues K100-D103. However, the chemical shift perturbation (CSP) of these residues was relatively lower in magnitude (<35 Hz) than the residues in the R17-M20 stretch. It was noticed that the residues K100-D103 are located at the end of the 6^th^ helix of the CH domain crystal structure^8^ closer to the first helix. Incidentally, when EB1n was titrated with ortho-phosphate instead of GTP, the chemical shift of K100 was perturbed to a small extent along with R17, which showed a larger perturbation. However, no perturbation was observed for H18 and other residues, which were affected by GTP (Supplementary Fig. S6), suggesting that the chemical shift change of K100 upon GTP addition could be due to an additional charge-charge interaction with phosphate. Therefore, the results infer that R17-M20 is the main GTP-binding site. Sequence analysis revealed that residues corresponding to the GTP-binding site of EB1 (RHD) are conserved among wide range of species (Fig. 2D). It should be noted that SRHD of EB1 is very much similar to NXRD consensus GTP-binding sequence of other proteins. Additionally, we also found that amino acid methionine (M) after SRHD is also conserved as shown by RHDM in colour in Fig. 2D).

**Figure 3:**
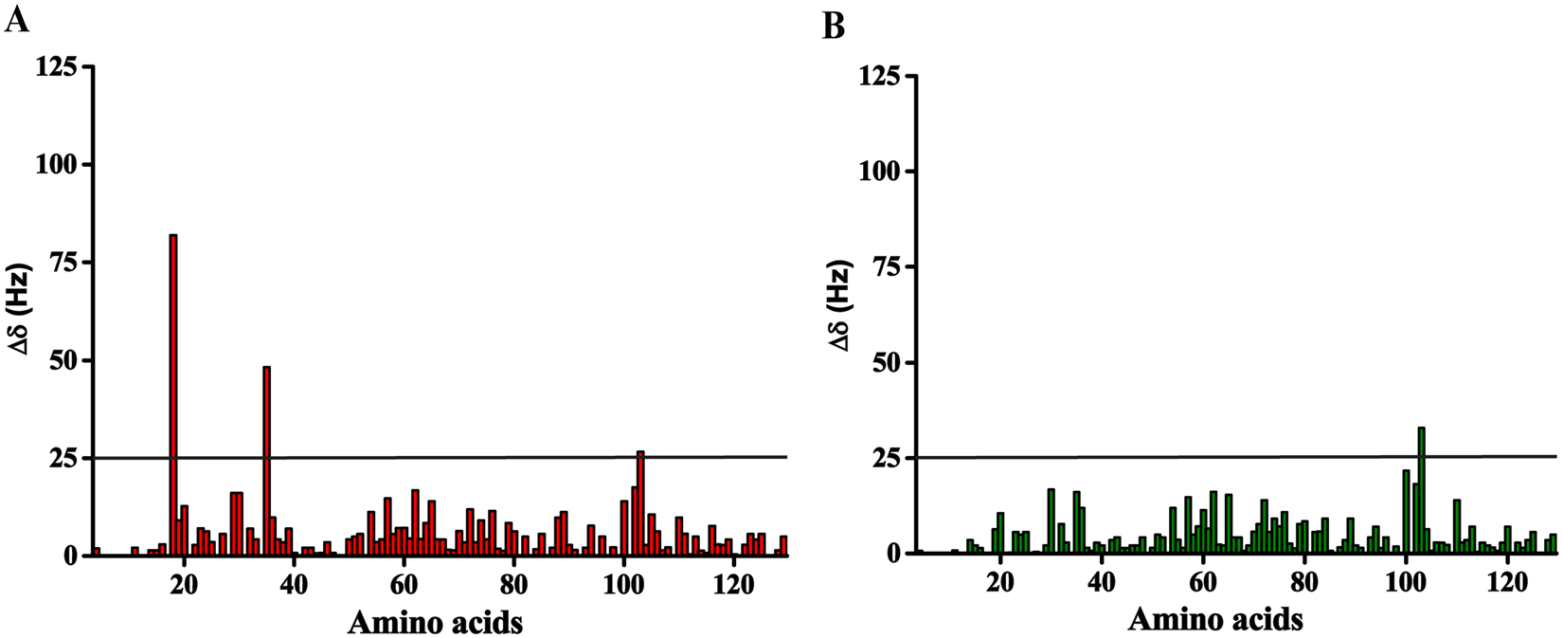
Double mutations of R17 and H18 in EB1n disrupt EB1n-GTP binding. (**A** and **B**) are bar plots showing the changes in chemical shifts of the amino acid cross peaks in the 2D ^1^H-^15^N HSQC spectra of EB1n R17A (0.1 mM) and EB1n R17A H18A (0.1 mM), respectively on addition of 25 mM GTP. The straight line represents a cut-off chemical shift change of 25 Hz. In (**A**) the chemical shifts of H18 and I35 were changed significantly upon GTP addition, indicating retaining of GTP binding, but to a lesser extent than EB1n-WT. In (**B**) the chemical shift changes of the amino acids were drastically reduced as compared to the wild-type protein, indicating almost complete loss of GTP-binding in EB1n R17A H18A. None of the amino acid residue of the double mutant showed chemical shift change above 25 Hz.

As R17 and H18 of EB1n show maximum chemical shift perturbations in the GTP-titration experiment (Fig. 2B), it is possible that these two residues are most critical for EB1n-GTP binding. To test this, we generated ^15^N-labeled single (EB1n R17A) and double mutants (EB1n R17A H18A) of EB1n. The peak positions of the residues in the HSQC spectrum of both the mutants were very similar to the wild type EB1n (Supplementary Fig. S7). This confirmed that the mutations did not lead to any significant global structural change in EB1n. We then analyzed the binding ability of the mutants with GTP by HSQC titration. The HSQC spectrum of the single mutant with GTP showed considerable reduction in the chemical shift changes of the amino acids, H18, D19 and M20 as compared to the wild-type EB1n. The lower values of chemical shift perturbation for the putative GTP-binding residues signify weakening of EB1n-GTP binding (Compare Fig. 3A with 2B). The I35 amino acid residue was also significantly affected by GTP titration with the mutant. Since I35 lies in close proximity to R17 in the crystal structure (Supplementary Fig. S5), mutation of R17 to alanine could have forced GTP to bind to the proximal I35 instead. The chemical shift perturbation of I35 in the single mutant was very similar to the R17 chemical shift perturbation in the wild type EB1n –GTP titration (compare Figs 2C and 3A). It is therefore conceivable that the role of R17 in the binding with GTP is taken over by I35 in the single mutant. It was also observed that the chemical shift perturbations of the same set of residues were less in the case of HSQC titration of EB1n with GDP as compared to GTP (Supplementary Fig. S8).

The effect of R17A H18A double mutation on the binding ability of GTP to EB1n was more severe. Almost no discernible changes in chemical shifts were observed when GTP (25 mM) was added to the double mutant (Compare Fig. 3B with Fig. 2B), indicating that the double mutation had almost completely abolished the GTP-binding ability of EB1n. ITC titration also showed no characteristic binding between R17A H18A EB1n and GTP (Supplementary Fig. S10). Taken together, results of the mutational study reveal that H18 is a key residue essential for GTP binding to EB1n.

### GTP binding suppresses the auto-inhibitory interaction in EB1

**Figure 4:**
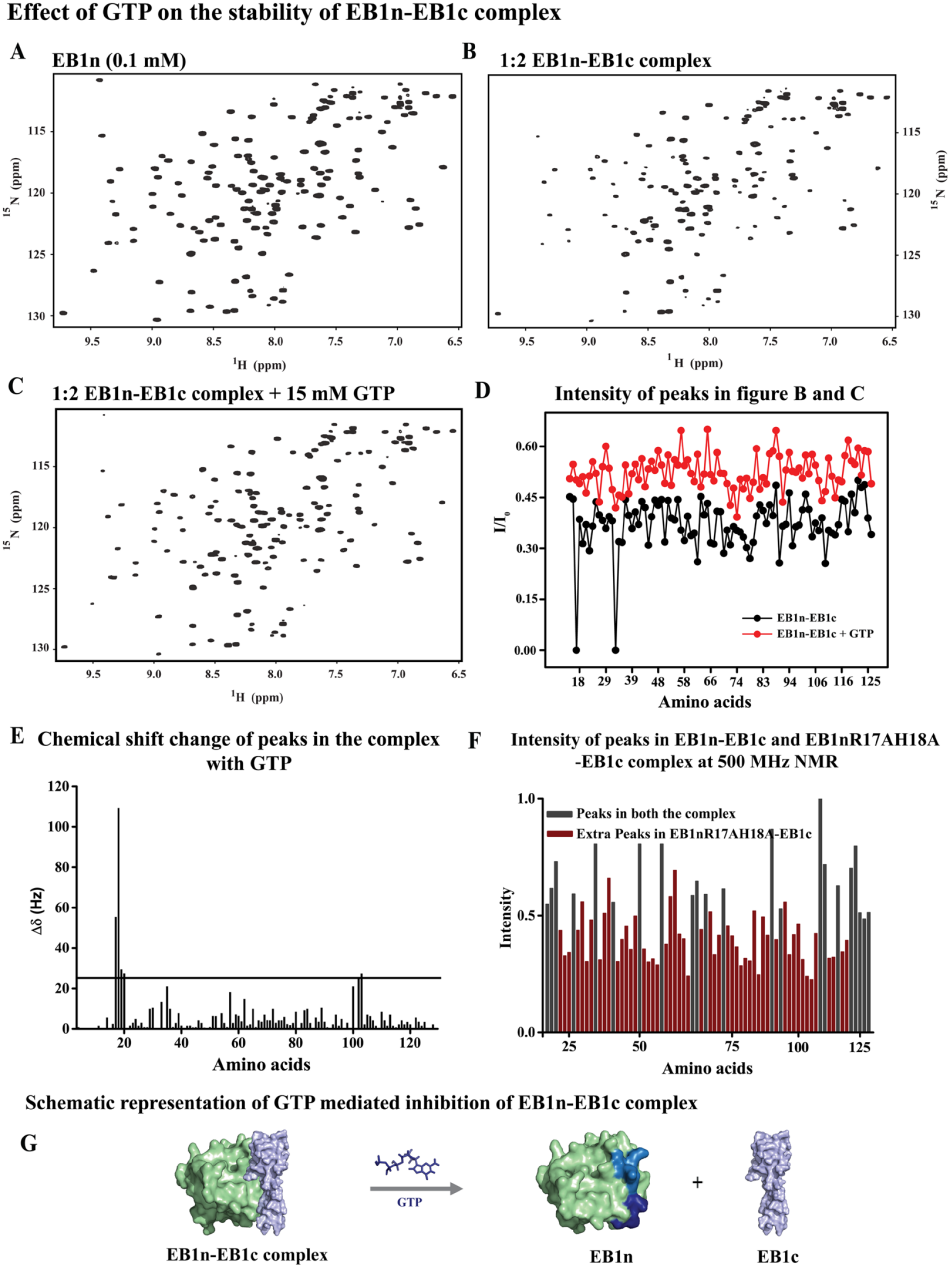
GTP binding suppresses auto-inhibitory interaction between EB1n and EB1c. Figures (**A**–**C**) show two-dimensional ^1^H-^15^N HSQC spectra of EB1n (0.1 mM), EB1n-EB1c mixture (1:2 complex), and EB1n-EB1c (1:2 complex) plus 15 mM GTP, respectively. The spectra were measured in 700 MHz NMR spectrometer. As only EB1n is ^15^N labeled, peaks of EB1n residues were visible. In the case of EB1n-EB1c without GTP, intensities of most of the EB1n peaks were reduced (compare **B** with **A**). NMR peak line broadening due to large size of the complex is attributed to the reduction in intensity. The peaks in C show the effects of GTP on the stability of EB1n-EB1c complex. In C, majority of the peaks that were broadened in the complex (shown in **B**) reappeared at 15 mM GTP, suggesting loss of interaction between EB1n and EB1c.The scaled intensities of peaks in the figure **B** (black) and figure **C** (red) is shown in Figure **D**. (**E**) Plot shows the chemical shift changes of EB1n residues in EB1n-EB1c mixture at 15 mM GTP. (**F**) The intensities of cross peaks in the HSQC spectrum of EB1n-EB1c (1 mM each) and EB1n R17A H18A-EB1c (1mM each) complex measured at 500 MHz NMR spectrometer. The peaks present in both the spectra were shown in grey bars and the red bars corresponding to intensities of extra peaks that appeared in the EB1n R17A H18A-EB1c complex. (**G**) Schematic representation of GTP-mediated inhibition of EB1n-EB1c interaction. Structural model of EB1n CH domain and EB1c were created from previously published crystal structures (EB1n CH: 1PA7; EB1c: 1YIG) by arranging them in pymol. Orientation of the domains is arbitrary and is not based on any experimental data.

Previous studies have shown that EB1 C-terminus (191–268, EB1c) can directly bind to the N-terminus (1–151, EB1n), independent of the intermediate loop region^26^. In order to probe the effect of GTP binding on EB1n-EB1c interaction, we performed the following NMR titration experiments. At first, we formed the adduct (EB1n-EB1c) by adding 2 equivalents of unlabeled EB1c (0.2 mM) to 1 equivalent of ^15^N labeled EB1n (0.1 mM). The intensity of the peaks in the resulting HSQC spectrum of EB1n in the complex was decreased compared to the HSQC spectrum of EB1n alone (Compare Fig. 4B with 4A). The reduction in intensity in Fig. 4B is the result of broadening of resonances associated with faster relaxation rate due to EB1n-EB1c complex formation. This is consistent with previous studies^26^. To this adduct, GTP (pH pre-adjusted to 6.9) was then added stepwise and the HSQC spectra were measured after each GTP addition. Almost all the peaks of EB1n showed increased intensity upon addition of 15 mM GTP (Fig. 4C). To quantify the changes, we took the ratio of intensity of the peaks of Fig. 4A and B. As shown by black points in Fig. 4D, the data clearly showed loss of peak intensities due to adduct formation. However, the intensity ratio of Fig. 4A and C increased significantly for almost all the residues (Fig. 4D, red points) indicating that addition of GTP resulted in destabilization of the EB1n-EB1c adduct. The loss of peak intensities in the HSQC spectrum of EB1n with addition of EB1c is owing to increased relaxation rate of nuclear spins because of the large size EB1n-EB1c complex. As the size of the molecule increases, the relaxation, which is reflected in the line width of the NMR peaks, also increases leading to broadening and eventual disappearance of the peaks. Consistently, we observed that the HSQC spectrum of the complex in the presence of 15 mM GTP resembled almost identical to that of the EB1n alone with GTP. These results indicate that GTP affects the same set of residues both in EB1n-EB1c complex and in EB1n. Therefore, the data illustrate that GTP interferes with the binding of EB1c to EB1n. Additionally, it also suggests that GTP and EB1c share the same binding pocket in EB1n.

Since the double mutant (EB1n R17A H18A) of EB1n does not bind to GTP (Fig. 3B), and that GTP and EB1c share the same binding pocket in EB1n, it is reasonable to think that the double mutation can also adversely affect the binding of EB1c to EB1n. Figure 4F shows the scaled intensity of HSQC spectra of 1 equivalent of EB1n R17A H18A mutant with 1 equivalent of EB1c (both black and red) compared to the mixture of wild type EB1n and EB1c (only black). Unlike the case of wild type EB1n-EB1c mixture, the intensities of the peaks from the HSQC spectra did not disappear upon adding the C-terminus to the mutant. Retention of the peaks in the HSQC spectrum indicates that EB1n-EB1c interaction was substantially impaired in the case of mutant. Taken together, the results demonstrate that the R17A H18A double mutation not only impairs the binding of GTP to EB1n but also impairs the binding of EB1 C-terminus to EB1n. Furthermore, GTP and the C-terminus share their binding sites in EB1n and the mutation leads to disruption of the binding of both GTP and EB1c to EB1n. This is shown in a representative model in Fig. 4G.

### Mutations of the GTP-binding sites in EB1 facilitate EB1 association with microtubules

**Figure 5:**
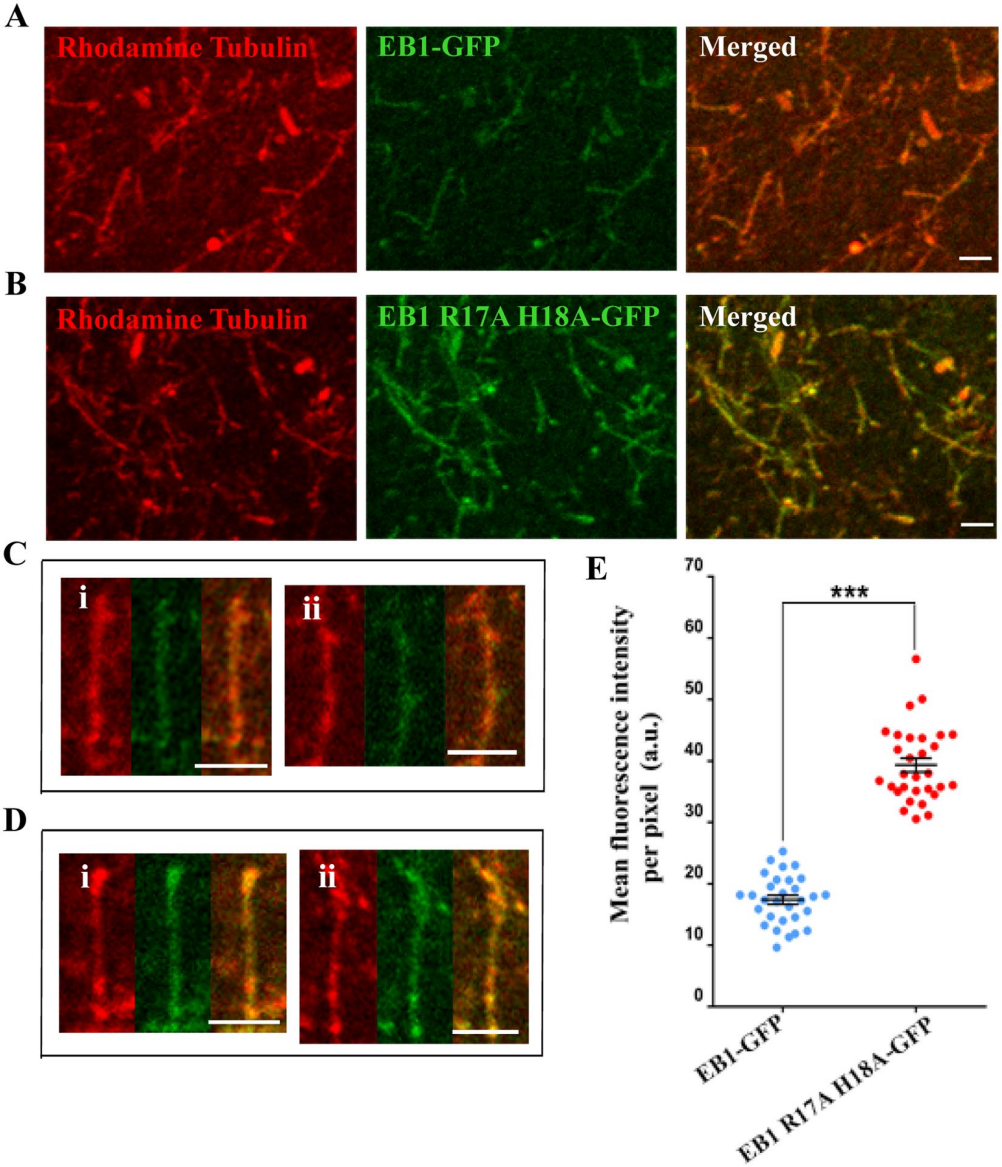
GTP-binding site mutation in EB1 facilitates EB1 binding to the microtubules. Rhodamine labeled microtubules added with (**A**) WT EB1-GFP or (**B**) EB1 R17A H18A-GFP mutant are shown. The representative images shown are single plane best-focused confocal images with the same display setting with a lower and higher grey values of 10 and 150, respectively for WT and mutant EB1 conditions. The enlarged images of single microtubules with WT EB1-GFP or mutant EB1-GFP staining are shown in (**C**, i, ii) and (**D**, i, ii), respectively. Scale bars are 2 μm. Data are representative of three independent experiments in which at least ~200 number of microtubules were imaged in each condition. (**E**). Plot shows the mean pixel intensity of WT EB1-GFP and the mutant EB1-GFP along the lengths of the microtubules. ***Refers to P value < 10^−10^ as determined by students t-test.

We next determined whether GTP-binding to EB1 has any effect on its ability to localize onto the microtubules. Since microtubule polymerization also requires GTP, it was not possible to selectively assess the effect of GTP on EB1 localization on the microtubules by directly adding GTP-bound EB1 to the microtubules. Instead, we took an alternative approach. As mutations of the GTP binding site residues disrupt interaction between EB1 N- and C-terminus (Fig. 4) and induces exposure of the microtubule-binding sites of the N-terminus^26^, we examined whether the mutations exert any effect on the localization of EB1 on the microtubules. Recombinant GFP fused wild-type or R17A H18A mutant EB1 was added to rhodamine-tubulin labeled microtubules that were polymerized in the presence of slowly hydrolysable GTP analog, GTPγS (Methods). Briefly, the microtubules after attaching to the glass coverslips were washed with fresh buffer to remove all the free GTPγS prior to add the EB1 proteins to the microtubules. Immunofluorescence imaging of the microtubules revealed that a relatively higher level of the mutant EB1-GFP is associated with the microtubules than the wild-type EB1-GFP (Fig. 5A–D). Intensity analysis showed ~2 fold increase of the intensity per pixel of the EB1 R17A H18A-GFP mutant bound to the microtubules as compared with the wild-type EB1-GFP (Fig. 5E).

**Figure 6:**
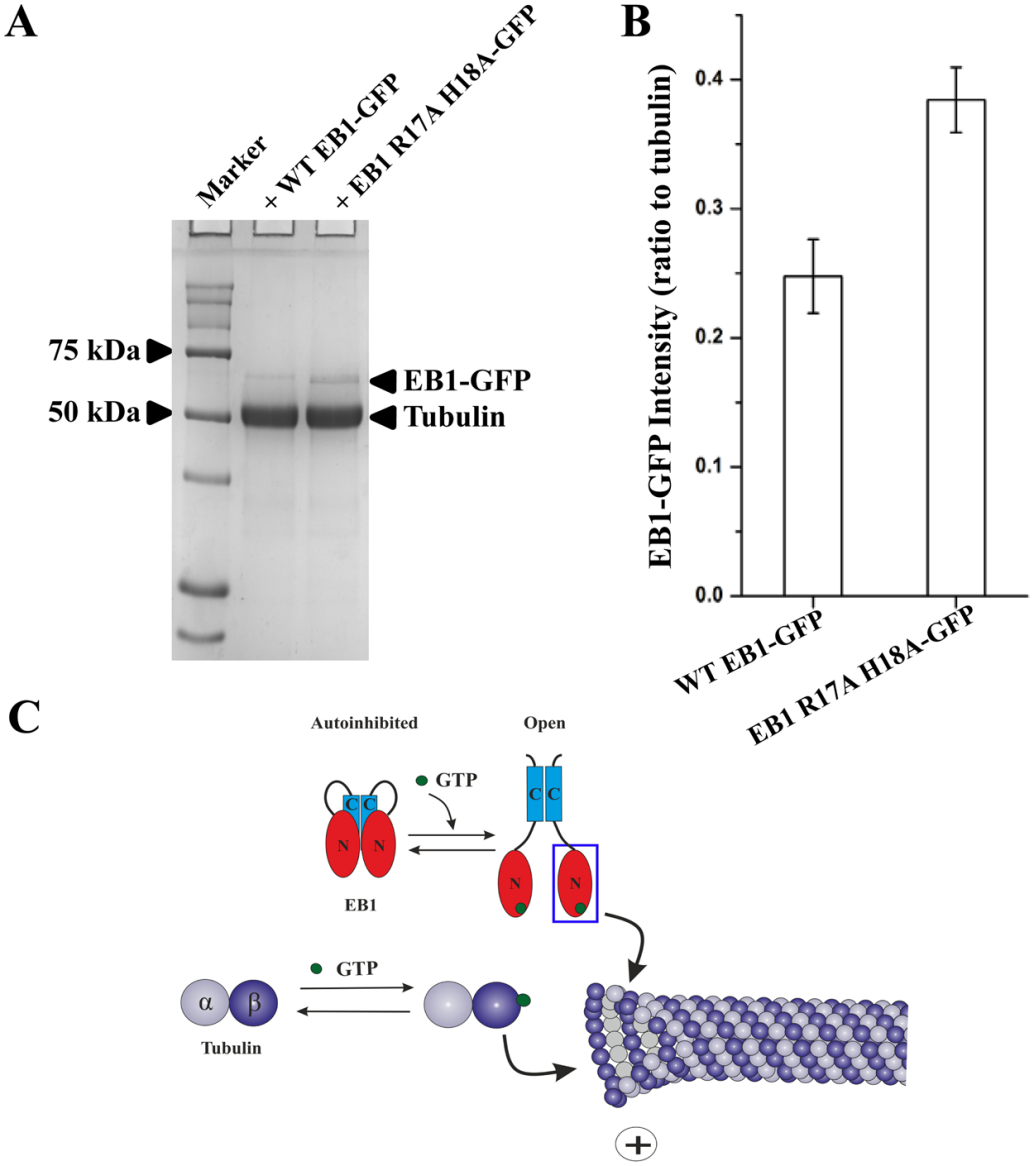
Biochemical analysis of wild-type EB1- vs. EB1 R17A H18A mutant- binding to microtubules. (**A**) Microtubules after polymerizing tubulin (40 μM) with 10% DMSO and 1 mM GTPγS onto glass coverslips, were extensively washed with buffer to remove free GTPγS and then WT EB1-GFP (10 μM) or EB1 R17A H18A-GFP (10 μM) was added to the coverslips. After 15 min incubation, the proteins were extracted from the coverslips by crushing the coverslips in SDS-PAGE sample buffer and the samples were analyzed by 10 % SDS-PAGE followed by Coomassie Blue staining. The full-length gel picture is provided in Supplementary Fig. S12. The gel image represents one out of three experiments. (**B**) Intensity plot of the amount of WT EB1-GFP vs. EB1 R17A H18A-GFP associated with microtubules based on the analysis of the protein bands in SDS-PAGE. Band intensities of EB1 proteins were normalized against tubulin band in each. Data are mean +/− SEM (based on three experiments). (**C**) A conceptual model of the possible regulation of EB1 function by GTP-binding. The full length EB1 dimer usually exists in an auto-inhibited conformation due to masking of its microtubule-binding sites by the interaction between its N- and C-terminus. Upon GTP binding, this intra-molecular interaction gets destabilized and the structure transforms to an open conformation, facilitating EB1-binding to the microtubules.

Microtubule association of EB1 R17A H18A mutant vs wild-type EB1 was further assessed by biochemical analysis. Microtubules were polymerized on glass coverslips under the same polymerization condition as of the imaging experiment in the presence of GTPγS. Wild-type EB1-GFP or EB1 R17A H18A-GFP was then allowed to bind to the microtubules attached on the coverslips after removing all the free GTPγS from the coverslips by washing with warm buffer (see Methods for details). SDS-PAGE analysis of total proteins extracted from the coverslips after washing off the microtubule-unbound proteins by a buffer wash showed presence of significantly higher amount of R17A H18A EB1-GFP associated with the microtubules as compared to the wild-type EB1-GFP (Fig. 6A). Quantification of the EB1 protein bands normalized against tubulin showed ~1.5 fold higher amount of EB1-mutant bound to the microtubules than the wild-type EB1 (three experiments) (Fig. 6B).

Our GTP-titration NMR results have shown that GTP destabilizes EB1n-EB1c binding (Fig. 4). As the EBH domain of EB1c suppresses the microtubule-binding ability of EB1n by binding to this domain in the full length protein^7^, it can be possible that GTP and the EB1 C-terminus share their binding sites in the EB1n domain. Supporting this possibility, we have shown that mutations of R17 and H18 to alanine in EB1n impair both GTP-binding and the binding of EB1c to EB1n (Figs 3B and 4F). Microtubule binding data also show that the mutations result in increased association of EB1 with the microtubules (Figs 5 and 6). Together, the results indicate that the GTP-binding site residues in EB1n are responsible for stabilizing EB1 in the auto-inhibited conformation and the inhibition can be released either by GTP binding to EB1n or by mutating the GTP-binding residues in EB1n; both of which negatively regulate EB1n-EB1c binding.

Free intracellular GTP concentration reported in earlier studies is between 100 μM to 1 mM^34,35^. This range of GTP concentration could be sufficient to saturate GTP-binding of EB1 considering the range of K_d_ reported here. It should be noted that heat change profiles of EB1n showed a slightly higher noise level in the ITC data. It could be due to small energy release associated with non-specific dynamic interactions of GTP with residues other than the residues of the main GTP-binding site. Also the heat change profile of EB1 full length did not exhibit complete saturation. This could be due to small change in monomer-dimer equilibrium of the protein at higher GTP concentration.

We also observed a noticeable difference in GTP-binding in NMR vs. ITC titration. It should be noted that ITC experiment was  performed in PIPES buffer without any salt, whereas NMR titrations were performed in phosphate buffer with high salt. This was needed in order to keep the protein soluble at high concentration, which is required for NMR. It was observed that buffer composition has a prominent role in contributing to this difference. For example, when we performed NMR titrations in HEPES buffer without any salt as compared to phosphate buffer in presence of salt, the K_d_ was reduced substantially from 19 mM to 3.8 mM (Supplementary Fig. S13A,B). However, the K_d_ obtained from NMR in HEPES was still higher than that of ITC. We think that EB1n-EB1n association at the high concentration of protein used in the NMR titrations could further contribute to the higher K_d_, since EB1n-EB1n association is expected to result in line broadening of the NMR peaks. The EB1n-EB1n interaction in the full length EB1 was reported earlier^33^. Specifically, the residues around K113 were shown to constitute the dimer interface. To understand if there is any influence of protein concentration in favouring association between EB1n molecules, proton line width of ^1^H-^15^N HSQC spectrum of EB1n was measured at two different protein concentrations (0.075 mM vs. 0.25 mM) in HEPES buffer (Supplementary Fig. S14). A 25% increase in line width was observed for most of the residues. Interestingly, consistent with the previously reported data^33^, F114 showed a very significant increase in line width. These findings indicate that the EB1n-EB1n association is favoured at higher protein concentration and thus, it may interfere in EB1-GTP binding. Because of low sensitivity of NMR peaks at low protein concentration, we could not further lower the protein concentration in the NMR titrations. In ITC, since the protein concentration was much lower (30 μM), the EB1n-EB1n association could be too weak to affect the K_d_ of EB1-GTP binding.

In the micro-environment of microtubules, tubulin is a major GTP-binding factor as the GTP-bound tubulin subunits are essential for polymerization of microtubules^36^. Recruitment of both tubulin and EB1 to the polymerizing ends of microtubules are crucial for proper assembly and dynamics of microtubules. Since EB1n, specifically the CH domain of EB1n is mainly involved in microtubule binding^8^, it is reasonable to think that similar to tubulin, the microtubule-binding ability of EB1n domain, so as the whole EB1 protein, could be controlled by GTP-binding. Supporting this idea, our microscopy and sedimentation data showed that the GTP-binding defective EB1 mutant associates with the microtubules at a relatively higher level than the wild-type EB1 (Figs 5 and 6). Stimulation of the microtubule-binding ability of EB1 mutant is probably due to increased exposure of the microtubule binding site residues of its CH domain. Similar to the effect of GTP-binding in the wild type EB1 protein, the mutation can relieve auto-inhibitory interaction between EB1 CH domain and EB1 C-terminus. As the GTP-binding site residues in the CH domain are also known to be involved in EB1-microtubule binding^7,8,26^, the increased exposure of those residues could allow more EB1-binding to the microtubules. Together, our findings suggest that GTP-binding activity of EB1 could be physiologically relevant. A conceptual model on possible regulation of EB1 function by GTP-binding is shown in Fig. 6C.

Sequence comparison of other known GTP-binding proteins in various organisms has indicated the presence of characteristic consensus sequences that are rich in both positive and negatively charged amino acids^37,38^. For example, translation activator human EF 1-alpha, RAS family proteins, Rho GTPases and G-proteins, they all consist of a GTP-binding site of consensus sequence NKXD (Gln-Lys-X-Asp). The amino acid sequence of the GTP-binding motif in EB1n as shown in this study (Figs 2, 3 and 4) is SRHD (Ser-Arg-His-Asp), which is very similar to the sequence NKXD (RHD of sequence RHDM shown in Fig. 2D). Therefore, the GTP-binding site in EB1 identified here does not seem to be unusual compared to the same in other GTP-binding proteins. As this motif is highly conserved among the EB1 homologs across species, the findings further indicate that the GTP-binding phenomenon might be conserved across species and they also point towards a possible molecular link of GTP binding with the cellular function of EB1, specifically its ability to target microtubules. Our microtubule-binding data support such possibility (Figs 5 and 6).

We compared the GTP binding region of EB1n with those of some known GTP-binding proteins, such as Rad G-domain-GTP analog complex, YchF complexed with GDP, and YsxC complexed with GDP^38–40^. In each of these proteins (Supplementary Fig. S15B,C and D), the phosphate group and the nucleotide part of GTP/GDP associate with distinct structural regions in the proteins. Similarly, our NMR chemical shift perturbation data showed that the affected residues upon GTP titration belong to two distinct regions in EB1n. The region 16SRHD19 is most likely associated with the nucleotide and the lesser-perturbed residues, 100K-D103 may be binding to the phosphate (Fig. 2C). However, it should be noted that the surface representations shown in Figs 2C and S5 and S15A are based on the crystal structure of free EB1n protein, not its GTP-bound form. Therefore, it may not represent the true bound structure and formation of a binding pocket in the actual GTP-bound form cannot be ruled out.

Earlier studies have shown that EB1-C terminus binds to the +TIP protein, p150^Glued^ -dynactin and facilitates EB1 localization onto microtubules^7^. It is thought that p150^Glued^ relieves EB1 from its EB1c-bound auto-inhibitory conformation and promotes its microtubule localization. The results of the present study indicate that GTP may exert a similar role. It stimulates microtubule localization of EB1 presumably by releasing it from the EB1c-bound auto-inhibited form. The results implicate that GTP-binding could be an additional mechanism in the activation of EB1 association with the microtubules. It will be interesting to determine in future how GTP-binding to EB1 affects its recruitment onto cellular microtubules and how it is linked to the p150^Glued^ -mediated regulation of EB1 auto-inhibition.

## Methods

### Materials

GTP, GDP, PIPES, HEPES, IPTG, EGTA, PMSF, DTT, protease inhibitor tablet and imidazole were obtained from Sigma (St. Louis, MO, U.S.A.). GTPγS [Guanosine-5′-o-(3-thiotriphosphate)] was procured from Roche Diagnostics, U.S.A. The reagents used for the preparation of minimal media such as FeCl_3_, ZnCl_2_, CaCl_2_, MnCl_2_, CoCl_2_. 6H_2_O were purchased from Himedia, India. MEM vitamin mixture and Na_2_EDTA were procured from life technologies, U.S.A. and AMRESCO chemicals, U.S.A. respectively. ^15^N labeled NH_4_Cl and ^13^C labeled glucose were obtained from Cambridge Isotope Laboratories, Inc., MA, U.S.A. Rhodamine-labeled tubulin was purchased from Cytoskeleton Inc. (USA.)

### Mutagenesis of single (R17A) and double (R17A H18A) EB1n (1–151) mutants

pET3d plasmid containing EB1n cDNA insert was used to construct the mutants. PCR reactions were carried out using Expand Long Template PCR System (Roche Applied science, Germany), which contains a mixture of Taq DNA polymerase and Tgo DNA polymerase. The two single-primer PCR products in each case were combined and denatured to separate the newly synthesized DNA strands from the plasmid template DNA. The sample was cooled gradually to allow re-annealing of the complementary strands. Non-mutated parental plasmid DNAs were digested with DpnI. The re-annealed mutant plasmids were transformed into competent DH5α cells. The mutations were confirmed by sequencing^41^.

### Plasmids and Proteins

The N terminus (1–151), EB1n cloned in pET3d vector (generously provided by Dr. Michel Steinmetz, PSI, SW) was expressed and purified as described previously^33^. Briefly, the EB1n plasmid was transformed into BL21 (DE3) cells and the cells were cultured under 1 mM IPTG induction for 5 hrs prior to lysis with the lysis buffer containing 20 mM HEPES, 2 mM PMSF, 0.5 mM DTT, protease inhibitor cocktail (Sigma Aldrich, U.S.A) followed by sonication and centrifuged at 30000 rpm at 4 °C for 1.5 hrs. The supernatant was loaded onto a PD-10 gravity flow column packed with SP Sepharose fast flow strong cation exchanger (GE Healthcare Life Sciences, U.S.A.), which was pre-equilibrated with 20 mM HEPES buffer, pH 7.5. The protein were eluted by using salt gradients formed by mixing 20 mM HEPES, pH 7.5 and 20 mM HEPES buffer with 1 M NaCl, pH 7.5 and the eluted fractions containing EB1n as verified by SDS-PAGE were collected and stored. About 100% pure EB1n was obtained. Similar methods were followed for the expression and purification of EB1n R17A H18A mutant. 6x His tagged human recombinant EB1c was cloned into pET28a vector (Novagen, U.S.A.) using the full length EB1 cDNA as template^32^. Briefly, the plasmid containing the 6xHis tagged C-terminus (191–268), EB1c was transformed into BL-21 (DE3) cells. Cells expressing the construct were cultured under induction with IPTG (1 mM) for 5 hours prior to lysis. The His-tagged protein was purified after passing the lysate through a Ni^2+^-NTA column (QIAGEN), followed by elution using 0.5 M imidazole. Protein concentrations were estimated using the Bradford method with BSA as the standard^42^. Full length His-tagged EB1 was purified as described previously^32^. Tubulin was purified as described earlier^43^.

### Protein Expression in Minimal Media

EB1n, or EB1n R17A or EB1n R17A H18A cloned in pET3d plasmid was expressed in *E*. *coli* BL21 (DE3) cells. ^15^NH_4_Cl and ^13^C-labeled glucose were used as sources of ^15^N and ^13^C in the bacterial culture media. The bacterial cells were first grown in LB medium until optical density (O.D_600_) of the culture reached 0.6. Then the cells were spun down and re-suspended either in minimal media containing ^15^NH_4_Cl to obtain the ^15^N uniformly labeled protein or in a media containing ^15^NH_4_Cl and ^13^C-labeled glucose for ^15^N and ^13^C double labeled protein. Bacterial cells in the minimal media were allowed to grow at 37 °C until O.D._600_ increased by 0.1 unit^44–46^ Protein expression was then induced by growing the cells at 1 mM IPTG for six hours. The pelleted cells were re-suspended with the lysis buffer, sonicated and then centrifuged at 30,000 rpm, 4 °C for 1.5 hrs to separate the supernatant from cell debris. The proteins in the supernatant were finally purified by cation exchange chromatography as described above for purification of unlabeled EB1n.

### Circular Dichroism (CD) Spectroscopy

Far-UV CD spectra of EB1n and EB1c were measured by using CD spectrophotometer (JASCO 815). A 0.2 cm path length cuvette was used for the measurements. CD spectra of the proteins (3 µM) in the presence and absence of gradients of GTP concentrations in PBS, pH 6.9 buffer containing 1 mM MgCl_2_ were measured in the 215–240 nm range. The respective concentrations of GTP alone were used as baseline during measurement of CD spectra for each sample.

### Isothermal Titration Calorimetry (ITC)

Thermodynamic measurements were performed using ITC-200 Microcalorimeter from Microcal (Northampton, MA, U.S.A). EB1n or full length EB1, after dialysis with 80 mM PIPES, 1 mM EGTA, 1 mM MgCl_2_, pH 6.9 (PEM buffer) was titrated at 25 °C against GTP, which was dissolved in the final dialysate. A typical titration involved injecting 20 injection volumes (2 µl) of GTP into the sample cell containing EB1 (201.6 µl) at 2.0 min intervals with continuous stirring^43^. The heat of dilution data corresponding to individual injections were analyzed using standard binding model with the system running Microcal origin 7.0 software. The ∆*H* and ∆*S* values were obtained using a non-linear least square fit of the data. The concentrations of EB1n and GTP used in three independent sets of experiments were 30 µM, 300 µM; 40 µM, 400 µM; and 15 µM, 150 µM, respectively. The same for EB1-GTP titrations were 20 µM, 200 µM; 20 µM, 200 µM; and 15 µM, 150 µM, respectively. EB1 concentration was calculated based on monomer. The data for EB1n-GTP corresponding to 30 µM EB1n, 300 µM GTP and the same for EB1-GTP corresponding to 20 µM EB1, 200 µM GTP are shown in Fig. 2. The K_d_ values and all the thermodynamic parameters were obtained by fitting the data points in the system-run Origin software based on non-linear least square fit. The final data points fitted for determining the parameters were obtained by subtracting the control data of GTP-titration to only the buffer from the raw data corresponding to GTP titration to the protein samples. The data points for EB1n –GTP titration were fitted without fixing the number of ligand binding sites. In the case of full length EB1–GTP titration, the data were fitted considering the number of binding sites as two as it allowed to obtain all the thermodynamic parameters with minimum errors.

### EB1n Resonance Assignment

Three dimensional NMR experiments HNCA, HNCACB and CBCACONH were recorded in Bruker Advance 500 MHz NMR spectrometer equipped with triple resonance probe for sequential assignments of EB1n. NMR samples were prepared in 50 mM potassium phosphate buffer (pH-6.9) containing 100 mM KCl, 1 mM DTT, 1 mM MgCl_2_ and 10% D_2_O. About 1 mM of EB1n was used for the measurement. All NMR experiments were processed by NMRPIPE^47^ and assigned using Sparky programme^48^. These assignments were compared with a previously published set of data^26^.

### NMR Titration Experiments

To determine interaction between EB1n and GTP and further identify the amino acids involved in the interaction, two-dimensional ^1^H-^15^N HSQC of EB1n was measured by adding increasing concentrations of GTP to it starting from 0.05 mM to 25 mM (0.05 mM, 0.5 mM, 1 mM, 2 mM, 5 mM, 10 mM, 15 mM, 20 mM and 25 mM). 0.1 mM ^15^N labelled EB1n in 50 mM potassium phosphate buffer containing 100 mM KCl, 1 mM DTT, 1 mM MgCl_2_ and 10% D_2_O was used for this experiment. A high concentration of GTP stock solution (250 mM) was prepared in 50 mM PBS buffer (pH-6.9). We have adjusted the pH of GTP stock solution to 6.9 and subsequently used for the NMR titration experiment. All ^1^H-^15^N HSQC spectra were acquired in 16 scans and 256 indirect points at 298 K. The titration experiments were repeated in the same way for the single mutated EB1n R17A and the double mutated EB1n R17A H18A. The GTP titration of EB1n, EB1n R17A and EB1n R17A H18A were performed in 700 MHz NMR spectrometer. To determine the role of GTP on the stability of EB1n-EB1c complex, EB1n (0.1 mM ^15^N labeled) was first mixed with the un-labeled EB1c (0.2 mM) at a molar ratio of 1:2 in 50 mM potassium phosphate buffer - pH 6.9 with 100 mM KCl, 1 mM DTT, 1 mM MgCl_2_ and 10% D_2_O and then ^1^H-^15^N HSQC spectra of EB1n-EB1c mixtures were measured before and after addition of GTP (15 mM). ^1^H-^15^N HSQC experiments were performed similarly for the mixture of EB1n R17A H18A mutant and EB1c using 500 MHz NMR spectrometer.

### Cloning and purification of recombinant EB1-GFP and EB1 R17A H18A- GFP

Full-length human EB1 cDNA cloned in pET22b eGFP plasmid (generously provided by Dr. Anne Straube, Warwick University, UK) with a C-terminal 6xHis-tag was used for expression and purification of the WT EB1-GFP. The same plasmid was used as a template to express and purify EB1 R17A H18A -GFP mutant. The plasmids were transformed and expressed in BL21 (DE3) cells and the proteins were purified by Ni^2+^-NTA affinity column. Protein concentrations were estimated using the Bradford method as described previously.

### Microtubule polymerization and imaging of microtubules

A mixture of 40 μM tubulin and 3 μM rhodamine-labeled tubulin was polymerized in presence of 10% DMSO and 1 mM GTPγS in BRB80 buffer (80 mM PIPES, 1 mM EGTA and 1 mM MgCl_2_), pH 6.9 for 15 minutes at 37 °C. The polymerized microtubule solution was diluted 500 fold with warm BRB80 buffer prior to sediment the microtubules onto Poly-Lysine coated glass coverslips, by ultra-centrifugation through 10% glycerol cushion at 22,000 rpm speed at 37 °C for 12 minutes. The microtubules attached to the coverslips were washed with warm BRB80 buffer to remove all the free GTPγS from the microtubules and the vicinity. 10 μM EB1-GFP or EB1 R17A H18A-GFP protein pre-incubated at 37 °C for 30 minutes was added onto the coverslips and incubated for another 10 minutes at 37 °C prior to fix the microtubules with 1% glutaraldehyde in warm BRB80 buffer. The glutaraldehyde-fixed microtubules on the coverslips were washed with warm BRB80 buffer and the coverslips were then mounted on glass slides using Prolong Gold antifade reagent. The microtubules were imaged using Leica SP5 laser scanning inverted confocal microscope. Imaging parameters were kept exactly same for the wild type and the mutant EB1 conditions.

### Biochemical analysis of microtubule binding

For biochemical analysis by sedimentation, tubulin (40 μM) was polymerized into microtubules in the presence of 10% DMSO and 1 mM GTPγS in BRB80 buffer by following the same experimental condition as of the imaging experiment described above and then the microtubules were sedimented onto Poly-Lysine coated glass coverslips by ultra-centrifugation through 10% glycerol cushion. The coverslips were then washed with warm BRB80 buffer to remove free GTPγS. 10 μM WT EB1-GFP or EB1 R17A H18A-GFP protein pre-incubated at 37 °C for 30 minutes was then added to the microtubules on the coverslips and incubated for another 10 minutes at 37 °C. Again after washing with warm BRB80 buffer to remove all the microtubule-unbound proteins from the coverslips, the microtubules and their bound proteins were extracted from the coverslips by sample buffer and the samples were run in 10% SDS-PAGE followed by Coomassie blue staining. Intensities of the protein bands were quantified by quantity-one software. The experiment was performed three times and the average + SEM was plotted based on the results of three experiments.

### Image Analysis

Immunofluorescence images were captured using a 100 X (1.4 N.A.) oil-immersion objective attached on a Leica SP 5 laser scanning inverted confocal microscope. The same image acquisition settings were used for both control and the mutant EB1-treated conditions. The images were analyzed using Leica Application Suite Advanced Fluorescence Lite software. To measure the wild type and the mutant EB1-GFP intensity on the rhodamine-labeled microtubules, region of interest (ROI) lines were drawn across the length of the individual microtubules and the GFP intensity was measured after subtracting the background of same area for each. A representative image showing the ROI is provided in the Supplementary Fig. S11. The resultant mean intensity per pixel of wild type EB1-GFP and the mutant EB1-GFP bound to the microtubules were plotted. About 30 microtubules were analyzed for each case to obtain the statistical significance.

### Data Analysis and Fitting

EB1 N-terminal structure shown in the manuscript is based on the published crystal structure data (PDB: 1PA7) using PyMOL^8^. Alignment of amino acid sequences of EB1 (1–80) of different species was performed by using Clustal Omega. R software was used for statistical analysis of the GFP intensity on the microtubules in the wild-type vs. mutant EB1-GFP condition. Shapiro-Wilk normality test was employed to check the distribution of the data. The normally distributed data were analyzed with modified Student’s (Welch) t-test at the 99% confidence level. SEM refers to standard error of the mean. The data were plotted and fitted using origin 8.1 or GraphPad Prism 6 software. The figures were arranged using Adobe Photoshop and Adobe Illustrator.

### Data Sharing Statement

All the data are available to share.

### Data availability

Structural data from PDB: No- 1PA7 (link: www.rcsb.org) were analyzed in the present study.

## Electronic supplementary material

Supplementary Data
