# Supplementary material for: GTP-binding facilitates EB1 recruitment onto microtubules by relieving its auto-inhibition
Source: Sci Rep. 2018 Jun 28;8:9792. doi: 10.1038/s41598-018-28056-y (PMC6023887; doi:10.1038/s41598-018-28056-y)
Supplement: Supplementary file 1 — Supplementary Data [file 41598_2018_28056_MOESM1_ESM.docx]

**Supplementary Information**

**GTP-binding facilitates EB1 recruitment onto microtubules by relieving its auto-inhibition**

K. K. Gireesh, A. Shine, R. Bhagya Lakshmi, Vinesh Vijayan, and Tapas K. Manna

**Supplemental Figure Legends**

**Figure S1:** SDS-PAGE followed by Coomassie blue-stained gel images of the fractions of purified (A) EB1n and (B) EB1c.

**Figure S2:** (A) The raw ITC data obtained from 20 injections of 250 µM GTP to 25 µM EB1c in PEM buffer at 25 ºC. Lower panel shows the non-linear least-square fit plot of the heat changes per mole of the added GTP as a function of molar ratio of GTP and EB1c. (B) The far UV CD spectra of EB1c (3 μM) in the absence (black) and presence of 0.5 (red), 1 (magenta) and 1.5 mM (green) of GTP. Insets in both B and C show the plots of CD values at 222 nm vs. GTP concentrations.

**Figure S3:** 2D ^1^H-^15^N HSQC spectrum of 0.1 mM EB1n. The spectrum was measured in Bruker 700 MHz NMR spectrometer with 16 scans and 256 indirect points at 298 K. 90% backbone N-H resonances from EB1n were assigned.

**Figure S4:** The overlaid 2D ^1^H-^15^N HSQC spectra of EB1n with increasing concentrations of GTP. The peaks in HSQC spectra of 0.1 mM EB1n in the absence of GTP are shown in red while the peaks from EB1n with 2 mM, 5 mM, 10 mM, 15 mM and 25 mM of GTP are shown in blue, green, yellow, cyan, pink and orange, respectively.

**Figure S5:** Three dimensional structure of EB1n (based on the crystal structure of EB1 1-131, PDB: 1PA7). The residues constituting the major GTP binding site, H18, D19, and M20 are shown by red sticks, while R17 is shown by grey stick. I35 shown in green lies in close proximity to the R17 in the crystal structure.

**Figure S6.** The chemical shifts changes of the amino acid cross peaks in the 2D ^1^H-^15^N HSQC spectrum of EB1n (0.1 mM in HEPES, pH-6.9) on addition of 25 mM orthophosphate. The spectrum was measured in 700 MHz NMR spectrometer.

**Figure S7:** An overlay of ^1^H-^15^N HSQC spectra of EB1n (red), EB1n R17A (blue) and EB1n R17A H18A (green). The peak positions of N-H resonances in the spectra are nearly the same, which indicates the absence of major structural changes in both the EB1n mutants compared with the wild-type.

**Figure S8**. Comparative chemical shifts changes of 0.1 mM EB1n after the addition of 25 mM GTP and 25mM GDP as measured at 700 MHz NMR spectrometer.

**Figure S9.** The upper panel shows raw isothermal titration calorimetry (ITC) data obtained from 20 injections of 150 µM GTP to 15 µM EB1n R17A H18A in PEM buffer at 25 ºC. Lower panel shows the heat changes per mole of the added GTP in the titration as a function of the molar ratio of GTP and EB1n R17A H18A.

**Figure S10.** Titration of the mixture of ^15^N-labeled EB1n and unlabeled EB1c with GDP followed by HSQC. (A-C) are two dimensional ^1^H-^15^N HSQC spectra of EB1n, mixture of EB1n and EB1c (1mM EB1n:1mM EB1c), mixture of EB1n and EB1c plus 30 mM GDP, respectively. In the EB1n-EB1c mixture, most of the peaks corresponding to the residues in EB1n are disappeared (Fig. B). Fig. (C) shows the effect of GDP on the stability of EB1n-EB1c complex. Only a very few peaks of EB1n reappeared with the addition of 30 mM GDP to the EB1n-EB1c mixture. (D) Scaled intensities of the cross peaks in the HSQC spectra of the EB1n-EB1c mixture and the mixture with GDP-added condition are shown. The black bars indicate the intensities of peaks in the HSQC spectrum of EB1n-EB1c mixture and the pink bars are the intensities of additional peaks that appeared while adding GDP to the mixture. The spectra were measured using 500 MHz NMR spectrometer.

**Figure S11.** Representative images of microtubules showing the regions of interest (ROI) used for intensity analysis. White and blue lines refer to microtubule and background, respectively.

**Figure S12.** Coomassie blue-stained full-length gel picture from which gel image for Fig. 6A was prepared. The three sets of lanes correspond to different volumes of the microtubule associated with EB1 WT and the mutant solutions loaded in the gels. Lane 1: molecular weight marker; lanes 2 and 3: 5 µl each of EB1 WT and EB1 mutant, respectively; lanes 5 and 6: 10 µl of each; lanes 8 and 9: 3 µl of each. The samples were loaded in different volumes to avoid any pipetting error. For simplicity, we have shown only the lanes 2 and 3 for the WT and mutant in the main Figure 6A. The results represent one out of three independent experiments.

**Figure S13. (A) and (B)** The change in the chemical shift and dissociation constant of H18 of EB1n (0.1 mM) in phosphate buffer (50 mM potassium phosphate, 100 mM KCl, 1 mM MgCl_2_ and 1 mM DTT at pH 6.9) and HEPES buffer (50 mM HEPES, 1 mM MgCl_2_ and 1 mM DTT at pH 6.9), respectively, upon GTP addition. Inset shows images showing the shift of peak position.

**Figure S14.** The line width in the proton dimension of peaks in the ^1^H-^15^N HSQC spectrum of EB1n at 0.075 mM (grey) and 0.25 mM (red) concentration. A 25% increase in line width was observed for most of the residues, indicating possible association between EB1n molecules.

**Figure S15.** (A) The crystal structure of the N-terminal domain (1-130) of EB1 showing the possible GTP-binding site (shown in blue)^1^. (B) Crystal structure of Rad G-domain bound to GTP Analog (shown in blue)^2^. (C) Crystal structure of protein, YchF bound to GDP (shown in blue)^3^. (D) Crystal structure of protein, YsxC bound to GDP (shown in blue)^4^.

**Figure S1**

**Figure S2**

**Figure S3**

**Figure S4**

**Figure S5**

**Figure S6**

**Figure S7**

**Figure S8**

**Figure S9**

**Figure S10**

**Figure S11**

**Figure S12**

**Figure S13**

**Figure S14**

**Figure S15**

**Chemical Shift Perturbation (CSP)**

The GTP binding site in EB1n was mapped from the chemical shift change of cross peaks in the ^1^H-^15^N HSQC spectrum of EB1n with the addition of GTP. Both the ^1^H and ^15^N chemical shift was used for calculating the magnitude of chemical shift change (∆δ). The equation used for combining ^1^H and ^15^N chemical shift difference is as follows

∆δ = $\sqrt{\left( {\Delta\delta}_{HN} \right)^{2}+\left( {0.17*\Delta\delta}_{N} \right)^{2}}$

∆δ = combined chemical shift in Hz

${\Delta\delta}_{HN}$ = chemical shift change in the ^1^H dimension (Hz)

${\Delta\delta}_{N}$ = chemical shift change in the ^15^N dimension (Hz)

**References**

1	Hayashi, I. & Ikura, M. Crystal structure of the amino-terminal microtubule-binding domain of end-binding protein 1 (EB1). *The Journal of biological chemistry* **278**, 36430-36434 (2003).

2	Sasson, Y., Navon-Perry, L., Huppert, D. & Hirsch, J. A. RGK family G-domain:GTP analog complex structures and nucleotide-binding properties. *Journal of molecular biology* **413**, 372-389 (2011).

3	Kukimoto-Niino, M., Murayama, K., Shirouzu, M., Kuramitsu, S., Yokoyama, S. Crystal structure of the GTP-binding protein YchF in complexed with GDP. *RCSB PDB*, doi:10.2210/pdb2DBY/pdb (2005).

4	Ruzheinikov, S. N. *et al.* Analysis of the open and closed conformations of the GTP-binding protein YsxC from Bacillus subtilis. *Journal of molecular biology* **339**, 265-278 (2004).
